# Structural Variation of Bamboo Lignin before and after Ethanol Organosolv Pretreatment

**DOI:** 10.3390/ijms141121394

**Published:** 2013-10-28

**Authors:** Yuan-Yuan Bai, Ling-Ping Xiao, Zheng-Jun Shi, Run-Cang Sun

**Affiliations:** 1 Beijing Key Laboratory of Lignocellulosic Chemistry, Beijing Forestry University, Beijing 100083, China; Emails: yuanhai_9@126.com (Y.-Y.B.); lingpingxiao@gmail.com (L.-P.X.); km-szj@163.com (Z.-J.S.); 2 State Key Laboratory of Pulp and Paper Engineering, South China University of Technology, Guangzhou 510640, China

**Keywords:** organosolv pretreatment, MWL, lignin, Py-GC/MS, HSQC NMR

## Abstract

In order to make better use of lignocellulosic biomass for the production of renewable fuels and chemicals, it is necessary to disrupt its recalcitrant structure through pretreatment. Specifically, organosolv pretreatment is a feasible method. The main advantage of this method compared to other lignocellulosic pretreatment technologies is the extraction of high-quality lignin for the production of value-added products. In this study, bamboo was treated in a batch reactor with 70% ethanol at 180 °C for 2 h. Lignin fractions were isolated from the hydrolysate by centrifugation and then precipitated as ethanol organosolv lignin. Two types of milled wood lignins (MWLs) were isolated from the raw bamboo and the organosolv pretreated residue separately. After the pretreatment, a decrease of lignin (preferentially guaiacyl unit), hemicelluloses and less ordered cellulose was detected in the bamboo material. It was confirmed that the bamboo MWL is of HGS type (*p*-hydroxyphenyl (H), vanillin (G), syringaldehyde (S)) associated with a considerable amount of *p*-coumarate and ferulic esters of lignin. The ethanol organosolv treatment was shown to remove significant amounts of lignin and hemicelluloses without strongly affecting lignin primary structure and its lignin functional groups.

## 1. Introduction

The energy crisis and climate change concerns caused by overuse of fossil fuels has led to a worldwide interest in sustainable biofuels [1]. Plant biomass, which is renewable and available in high amounts and relatively low cost, is an ideal source of sustainable energy and biobased products [2]. Most plant biomass is lignocellulosic and mainly consists of three biopolymers: cellulose, hemicelluloses, and lignin, which together form a complex and rigid structure [3].

Lignin is a complex aromatic heteropolymer that forms a matrix with hemicelluloses and together they account for 18%–40% of the total dry weight of the plant [4]. The heterogeneous and highly cross-linked macromolecule of lignin is built up of different inter-unit linkages, such as β-*O*-4, β-β, β-5, β-1, 5-5, 4-*O*-5, *etc.* [5]. Moreover, lignin is linked by ether bonds between phenyl-propane units, which are not readily hydrolysable [6].

Among biomass feedstocks, Bamboo *Dendrocalamus brandisii*, belonging to *Bambusoideae* of *Gramineae*, has strong and abundant woody stems and is mainly distributed in southeast Asia including the southwest region of China [7]. Because of its easy propagation, fast growth, and high productivity, *D. brandisii* is considered one of the most potential non-wood forest feedstocks to replace wood resources. Various studies have been concerned with the lignin of bamboo material. However, the isolation, purification, and high-value application of lignin is still a challenge so far. Deriving lignin from the bamboo using organosolv fractionation processes might be a good choice to use the material as a fiber resource, if lignin and its derivatives can be obtained with added value when compared with that of similar synthetic compounds derived from oil.

Different kinds of physical and/or chemical pretreatments have been proposed for the conversion of wood and agricultural wastes [8]. Among these methods, the ethanol organosolv process is promising, since it allows clean fractionation of the biomass. Ethanol organosolv pretreatment allows an efficient fractionation of the raw material into a cellulose rich residue, a water soluble fraction mainly containing hemicellulosic sugars and large quantity of organosolv lignin fraction. Moreover, this process converts the recalcitrant lignocellulosic matrix to a reactive cellulosic substrate that can be readily digested by cellulase, resulting in a high conversion of cellulose to glucose [9]. Organosolv lignins are high-purity, low molecular weight and sulfur-free products. In addition, they are soluble in many organic solvents, possess low glass transition temperatures, and are easier to thermally process than Kraft lignins [10]. The knowledge of the structural changes imparted by pretreatments to carbohydrate and lignin structures can provide valuable information to understand the pretreatment mechanism and, thus, contribute to the improvement of current approaches or the development of new pretreatment methods [11]. Ethanol organosolv process is normally operated at a high temperature (>150 °C) with or without the addition of catalysts.

Recently, the conversion of ethanol organosolv lignin (EOL) to a potential fuel precursor for green gasoline/diesel by catalytic hydrogenolysis has been demonstrated. Generally, milled wood lignin (MWL) is considered to be representative of the original lignin but it usually gives low yields and contains a significant amount of carbohydrate contamination [12]. Cellulolytic enzyme lignin (CEL) was found to be structurally similar, but it has a higher yield, so it is more representative of the total lignin in wood than MWL. The carbohydrate amount of CEL is lowered by cellulolytic enzyme treatment before solvent extraction [13]. However, all these methods usually require intensive ball milling of the biomass for a period of hours to weeks [13].

The aim of the present work was to elucidate the changes produced in the structure and composition of bamboo lignin before and after organosolv pretreatment. For this purpose, lignin from bamboo in native form, in fractionated form after organosolv pretreatment, and enzymatic pretreatment were thoroughly investigated. These lignin fractions have been extensively analyzed by using several spectroscopic and chromatographic non-destructive techniques, including ^13^C nuclear magnetic resonance spectroscopy (NMR), heteronuclear single-quantum coherence (HSQC-NMR) spectroscopy, Fourier transform infrared (FT-IR), and gel permeation chromatography (GPC). Moreover, the untreated and pretreated bamboo materials were characterized by solid-state cross-polarization/magic angle spinning (CP/MAS) ^13^C NMR and pyrolysis-gas chromatography/mass spectrometry (Py-GC/MS).

## 2. Results and Discussion

### 2.1. Sugar Analysis

The results of component analysis of the original and pretreated bamboo, and the carbohydrate analysis of the isolated lignin samples are summarized in Table 1. The composition of untreated bamboo was determined to be 47.2% cellulose, 23.9% hemicelluloses, 23.8% Klason lignin, 1.5% acid-soluble lignin, and 1.4% ash on a dry weight. This result agrees well with recent analysis results reported by Shi *et al.* [14]. Compared with the raw material, the glucan of ethanol organosolv pretreated bamboo increased notably (52.3%) but the Klason lignin decreased (17.3%). The results of chemical analysis of the four isolated lignins indicated that glucan and xylan were the predominant carbohydrates and mannan was found only in very small amounts. Compared with MWL_u_, the polysaccharides in EOL showed lower amounts of glucan, and relatively higher amounts of arabinan, xylan and galactan, indicating the removal of a large amount of the hemicellulosic content from the solid phrase during the treatment. This result was in accordance with the amount of sugar contents of MWL_p_. For CEL, the removal of carbohydrates using cellulase increased the yields of lignin in the following aqueous dioxane extraction [13,15], which was ascribed to the inclusion of lignin released from its physical and/or chemical association with carbohydrate by the enzyme treatment. Therefore, the CEL includes not only the lignin normally isolated as MWL, but also lignin, which was associated with carbohydrates, giving rise to the relatively higher content of carbohydrates [13]. REL contained two main sugars, which were glucan (17.3%) and xylan (4.5%), and this could be explained by the incorporation of the cellulose and hemicelluloses during the enzymatic treatment [16].

### 2.2. Pyrolysis-Gas Chromatography/Mass Spectrometry

Py-GC/MS is a powerful tool for the *in situ* characterization of plant constituents. The material is pyrolyzed to produce a mixture of relatively simple phenols, which come from the cleavage of ether and carbon-carbon linkages. These phenols retain their substitution patterns from the lignin polymer, and it is thus possible to identify compounds from the H, G, and S lignin units [17].

**Figure 1:**
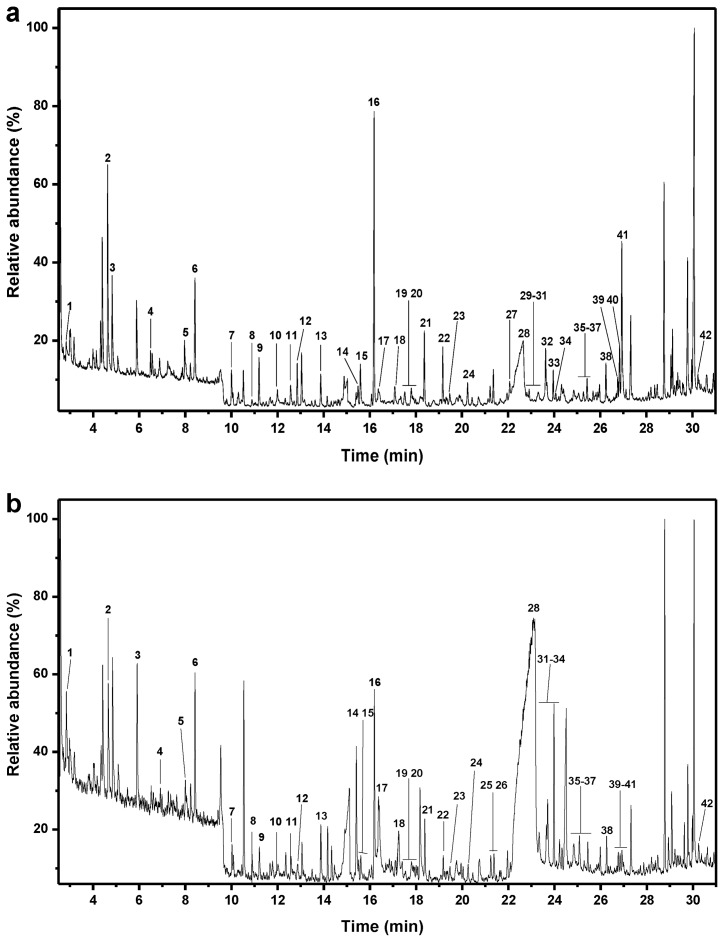
Pyrogram of (**a**) untreated and (**b**) ethanol organosolv pretreated bamboo. The structures of the labeled compounds are shown in Figure 2.

**Figure 2:**
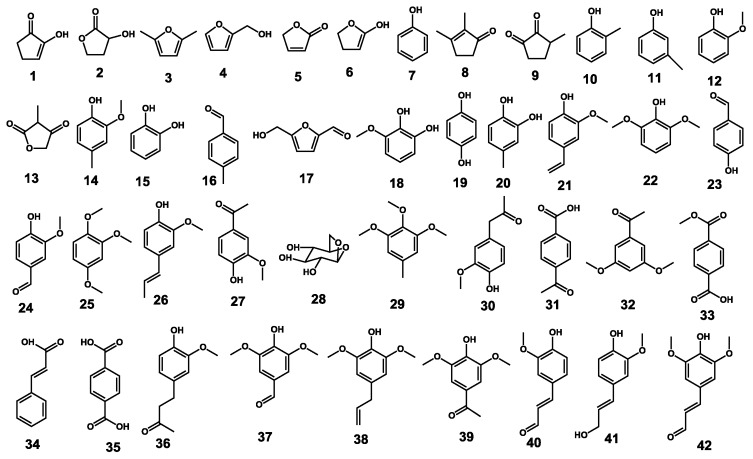
Compound structures. Assignments of all the structural compounds are labeled in Figure 1.

The pyrograms of the untreated and pretreated bamboo and the identified compounds are shown in Figures 1 and 2. The identification and relative molar abundances of the released lignin breakdown products are shown in Table 2. Relative peak areas were calculated for pyrolysis products from phenylpropanoid compounds (including guaiacyl (**G**) and syringyl-type (**S**) phenols), and the total areas of the peaks were normalized to 100% [18]. The pyrograms (Figure 1) show a series of products characteristic of pyrolysis of phenylpropanoid compounds in both untreated and pretreated bamboo. The main pyrolyzed products are G lignin derivatives (peaks **10**, **12**, **15**, **27**, **40**, **41**) and S lignin derivatives (peaks **18**, **22**, **32**, **38**).

In the case of the untreated bamboo sample, the estimated S/G ratio of the aromatic fraction was 0.4; in contrast, the S/G ratio calculated for the pretreated material sample was 0.6. The result revealed a decreased G-derived lignin content in the pretreated bamboo sample, indicating a preferential lignin degradation site at the G-units.

### 2.3. FT-IR Spectra

**Figure 3:**
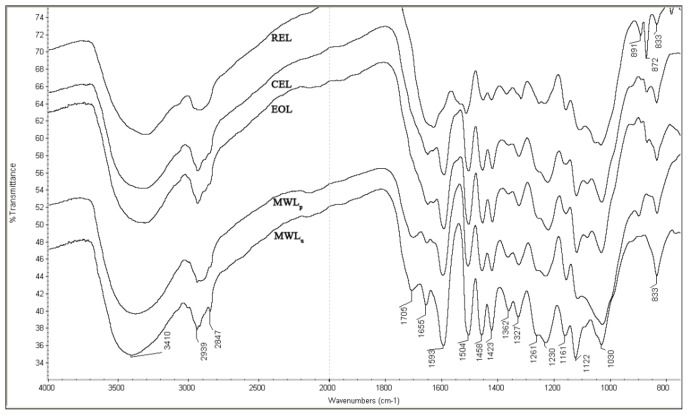
FT-IR of the lignin fractions.

FT-IR spectroscopy was further used to analyze the structural changes of the lignin fractions. The spectra and assignments are shown in Figure 3 and Table S1 in the Supplementary material. Despite some differences, MWL_u_, MWL_p_, EOL, and CEL spectra profiles are rather similar as a whole, indicating a comparable chemical structure of those extracted lignins. They all showed strong hydroxyl bond (O–H) stretching at 3401 cm^−1^ and C–H stretching vibrations at 2939/2847 cm^−1^. The bands between 1790 and 1680 cm^−1^ are characteristic of carbonyls. The bands at 1705 cm^−1^ and 1655 cm^−1^, observed in MWL_u_, are assigned to carbonyl stretching in unconjugated ketones and conjugated carbonyl groups, which can be mainly attributed to the coumaryl ester group, respectively. The intensity of these two bands decreased from the spectra of MWL_u_ to MWL_p_ to EOL and to CEL, and almost disappeared in that of REL. In addition, the intensity of the band at 1362 cm^−1^ showed the same tendency. It originates from the aliphatic C–H stretch in methyl (not in methoxyl) and phenolic hydroxyl groups. The bands at 1593, 1504, and 1423 cm^−1^ arise from the aromatic skeletal vibrations. The absorption at 1458 cm^−1^ is attributed to the C–H asymmetric deformations. The signal of the typical C–H band of acetyl methyl group observed at 1362 cm^−1^ in MWL_u_ was stronger than that of other lignin fractions. The absorptions at the wavelengths of 1327 and 1122 cm^−1^ correspond to syringyl units and those at around 1261 and 1161 cm^−1^ belong to guaiacyl units. As compared with MWL_u_, a decrease in intensity was observed at 1122 cm^−1^ of MWL_p_, which is assigned to aromatic skeletal and C–O stretch [19]. The band at 1030 cm^−1^ is attributed to aromatic C–H in-plane deformation vibrations, and the absorption at 833 cm^−1^ is due to C–H out-of-plane stretching [20]. The spectra of REL exhibited typical absorptions at 1152 cm^−1^ which was attributed to the association of xyloglucan. The absorption at 891 cm^−1^ is a typical absorption of the obstinate cellulose during the enzymatic treatment. This finding is consistent with the results obtained from the sugar analysis. Because the C=O vibrations cause a band at around 1270 cm^−1^, the absorbance here is higher than in the case of standard GS spectra. Another important spectral feature of HGS lignin is the intense band at 833 cm^−1^ (the aromatic C–H out of plain vibrations in H unit). Moreover, the presence of the band at 1161 cm^−1^ always permits a clear assignment to the HGS type [21].

### 2.4. Molecular Weight Distribution

Lignin samples are only slightly soluble in tetrahydrofuran (THF), a common solvent used for gel permeation chromatography (GPC). Therefore, the ball-milled lignin fractions were acetylated using acetic anhydride/pyridine. The values of the weight-average (M_w_), number-average (M_n_) molecular weights and the dispersity (M_w_/M_n_) of the lignin fractions are displayed in Table 3[1]. Lignins are highly branched heterogeneous materials, and polystyrene equivalent “molecular weights” are only rough indications of molecular size based on hydrodynamic volume. The molecular weight of MWL_u_ was smaller than that of MWL_p_ in the acetylated form, and the EOL extracted after ethanol organosolv pretreatment exhibited a decrease in molecular weight but an increase of the dispersity index. This could be due to the higher solubility of low-molecular-weight lignins with branched and cross-linked structures in the ethanol/water solvent. However, the condensed lignin was much more difficult to be fractionated or get it dissolved in the pulping processes [11]. In addition, all lignin fractions possessed relatively narrow molecular weight distributions, as shown by M_w_/M_n_ < 3.

### 2.5. HSQC NMR Spectra

**Figure 4:**
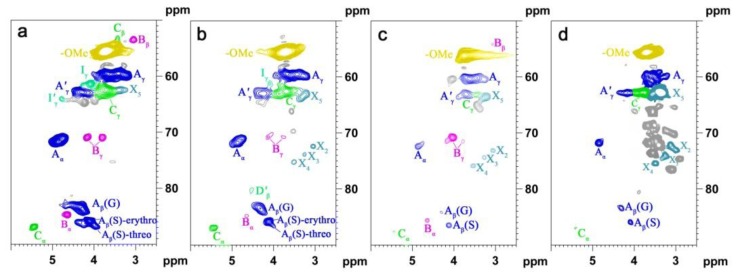

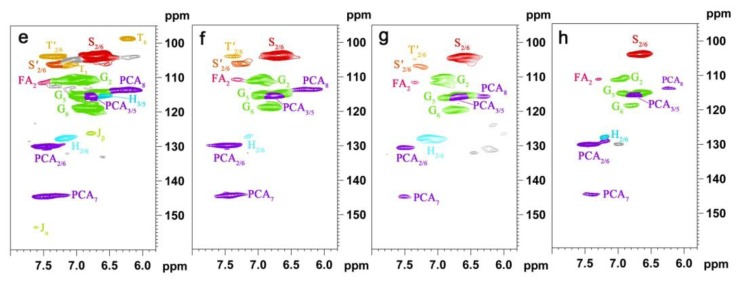
Side-chain (δ_C_/δ_H_ 50–90/2.5–6.1) region in the HSQC NMR spectra of (**a**) MWL_u_; (**b**) MWL_p_; (**c**) EOL and (**d**) CEL; Aromatic (δ_C_/δ_H_ 95–160/5.8–8.0) region in the HSQC NMR spectra of (**e**) MWL_u_; (**f**) MWL_p_; (**g**) EOL; and (**h**) CEL.

**Figure 5:**
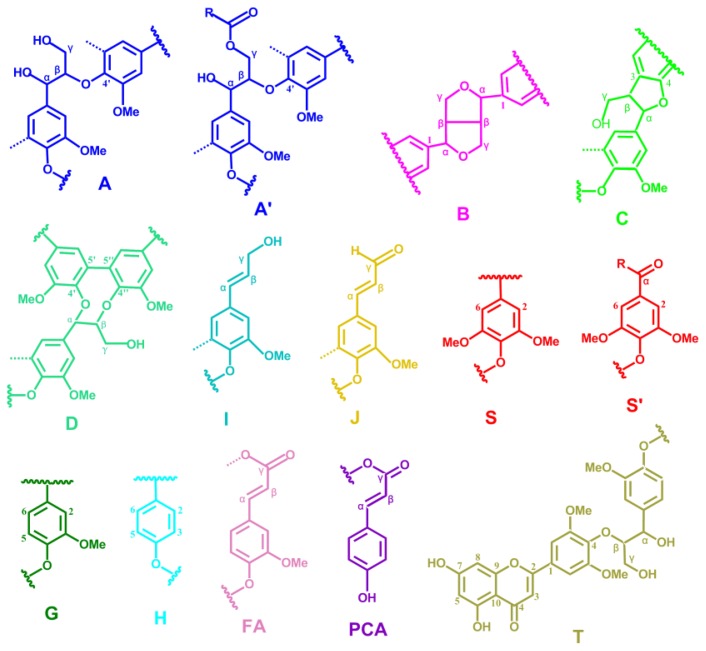
Main substructures present in the lignin fractions of bamboo (*D. brandisii*), as revealed as 2D HSQC NMR: (**A**) β-*O*-4′ substructures; (**A′**) β-*O*-4′ substructures with acylated γ-OH; (**B**) resinol substructures formed by β-β′ coupling; (**C**) phenylcoumaran substructures formed by β-5′ coupling; (**D**) spirodienone substructure formed by β-1′ coupling; (**I**) cinnamyl alcohol end-groups; (**J**) cinnamyl aldehyde end-groups; (**PCA**) *p*-coumarate units; (**FA**) ferulate units; (**H**) *p*-hydroxyphenyl units; (**G**) guaiacyl units; (**S**) syringyl units; (**S′**) oxidized syringyl units bearing a carbonyl at C_α_; (**T**) a likely incorporation of tricin into the lignin polymer through a G-type β-*O*-4′ linkage.

In order to get additional information on the lignin structure, bamboo lignin samples, which were obtained from different isolation procedures, were analyzed by 2D NMR. The lignin spectra are deposited in Figure 4, and the main lignin correlation assignments are presented in Table 4 by comparing with the literature data [2,22–26]; the main substructures are illustrated in Figure 5.

In the side chain region of lignin, the intense signals showed the presence of the major interunits linkages including β-*O*-4′ aryl ether (structure **A**), resinol (structure **B**), phenylcoumaran (**C**), and spirodiene structures (structure **D**) and so on. The C–H correlations in structure **A** were observed for α- and γ-C positions at δ_C_/δ_H_ 72.4/4.85 and 60.1/3.22 ppm, respectively. HSQC analysis demonstrated that MWL_p_ and EOL had a lower signal intensity of β-*O*-4′ linkage when compared with MWL_u_. El Hage *et al.* [27] suggested that the scission of β-*O*-4′ linkages was the major mechanism of lignin breakdown during organosolv pretreatment of lignin from *Miscanthus × giganteus*. The β-correlations from β-aryl ether units clearly separate into these respective G and S types, namely, **A_β_(G)** and **A_β_(S)** and confirmed at δ_C_/δ_H_ 83.6/4.30 and 85.8/4.10, respectively. The spectra showed the presence of intense signals at δ_C_/δ_H_ 62.8/4.28 corresponding to the γ-C/H of γ-acylated units (structure **A**′**_γ_**). Therefore, the HSQC spectra implied that these lignins were extensively acylated at the γ-position of the lignin side chain. Structure **B** was evidenced by C–H correlations at δ_C_/δ_H_ 84.7/4.65, 53.5/3.05, 71.0/4.17 and 70.9/3.80 ppm for C_α_–H_α_, C_β_–H_β_, and C_γ_–H_γ_, respectively. The presence of structure **C** was verified by its C/H correlations for α-, β-, γ-C positions at δ_C_/δ_H_ 87.1/5.45, 53.2/3.43, 62.4/3.71 ppm, respectively. Small signal corresponding to structure **D** could also be observed in the spectrum (at contour levels lower than those plotted), its C_β′_–H_β′_ correlations being at δ_C_/δ_H_ 80.3/4.54. Minor amounts of cinnamyl alcohol-end groups (**I_γ_**) could also be detected in the HSQC spectrum of the untreated MWL, as revealed by the C_α_–H_α_ correlations at δ_C_/δ_H_ 61.4/4.09. In the lignin spectra (Figure 4b–d), a dramatic decrease in side chain linkages was observed, and the corresponding cross-signals showed very low intensities and were even absent. All of these results indicated the extensive breakdown of β-*O*-4′ linkages during the ethanol organosolv treatment.

The aromatic ^13^C–^1^H region offers information on lignin constitutive units (such as **S** and **G** units), some of them bearing oxidized side chains. The cross-peaks for **S_2/6_** and **G_2_** were observed at δ_C_/δ_H_ 104.3/6.70 and 111.1/6.97, respectively. Signals for C_2,6_–H_2,6_ of **H** units at δ_C_/δ_H_ 128.0/7.17 were also detected in the HSQC spectra, although in lower amounts. This confirmed that the H-unit content in the lignins from these bamboo samples, similarly as in other grasses, was quite low (~3%, Table 5). Signals corresponding to C_2,6_–H_2,6_ correlations in C_α_-oxidized S-lignin units (**S′**) were observed at δ_C_/δ_H_ 106.3/7.30. The G units displayed different correlations for C_2_–H_2_, C_5_–H_5_ and C_6_–H_6_ as **G_2_**, **G_5_**, and **G_6_**. Small signals correspond to C_α_–H_α_ correlations (at δ_C_/δ_H_ 153.5/7.61 ppm) and C_β_–H_β_ correlations (at δ_C_/δ_H_ 126.2/6.79) of cinnamyl aldehyde end-groups (**J**). Prominent signals corresponding to *p*-coumarate (**PCA**) were observed in the spectra of MWL_u_. Cross-signals corresponding to the C_2,6_–H_2,6_ at δ_C_/δ_H_ 130.2/7.46 and C_3,5_–H_3,5_ at δ_C_/δ_H_ 115.4/6.76 correlations of the aromatic ring and signals for the correlations of the unsaturated C_7_–H_7_ at δ_C_/δ_H_ 144.5/7.43 and C_8_–H_8_ at 113.6/6.26 of the *p*-coumarate unit were recognized in the region of the HSQC spectra. Signals corresponding to the C_2_–H_2_ correlations of ferulate moieties **(FA**) were also observed at δ_C_/δ_H_ 111.5/7.49 in the spectra [28]. The correlations corresponding to the unsaturated C_α_–H_α_ overlapped with those of the *p*-coumarate. Tricin (**T**) is considered a dominant flavones in cereal crop plants, and mainly detected in leaves and stems [29]. It is widely distributed in grasses, including wheat, rice, barley, sorghum, and maize, and can occur in either free or conjugated form. Its signals appeared in the HSQC spectrum at δ_C_/δ_H_ 103.9/7.32, 106.1/7.04, and 98.8/6.22 corresponded to the C′_2,6_–H′_2,6_, C_3_–H_3_, and C_2,6_–H_2,6_ correlations, respectively [30].

Substantial structural changes were observed when comparing the HSQC spectrum of MWL_p_ EOL and CEL with the MWL_u_, where the presence of a greater number of signals and broader signals implied more complicated lignin structures after the fractionation processes. For MWL_p_, a characteristic is the absence of signals corresponding to the **C_β_** and **B_β_**, suggesting the degradation of β-aryl ether and resinol. Lignin degradation was also apparent as a result of the disappearance of **D_β_**_′_, **B_α_**, **FA_2_**, **H_2/6_**, **J_α_**, and **J_β_** cross-peaks, and the decreased intensities of **S** and **G** correlations. The aromatic area was almost identical for both MWLs from the original and treated bamboo. Interestingly, the spectrum of MWL_p_ showed predominant carbohydrate cross-signals (**X_2_**, **X_3_**, and **X_4_**), which partially overlapped with some lignin moieties. The EOL and CEL displayed the same features which may account for the signal expression of some degraded monosaccharide. As shown in the spectra in Figure 4, it was obvious that the isolated CEL contained significant amounts of carbohydrates as colored in grey in the spectrum. The EOL spectra in the side chain region showed the disappearance of the intensity of the peaks corresponding to **C_β_**, **I_γ_**, and **D_β_**_′_, validating the degradation of β-aryl ether, cinnamyl alcohol, and spirodienone units.

The relative abundances of the main lignin interunit linkages and end-groups, as the molar percentage of the different lignin units (**H**, **G**, and **S**), *p*-coumarates, and ferulates, as well as the molar S/G ratios of the lignin in bamboo, estimated from volume integration of contours in the HSQC spectra, are shown in Table 5. With respect to the different linkage types, MWL_u_ showed a predominance of β-*O*-4′ aryl ether linkages (**A**, 89.4% of the total side chains) followed by β-β′ resinol-type units (**B**, 5.5%) and a lower amount of β-5′ phenylcoumaran substructures (**C**, 5.1%). As compared with MWL_u_, MWL_p_ demonstrated a lower relative proportion of β-*O*-4′ and β-β′, which resulted in a higher relative proportion of β-5′ phenylcoumaran substructure. The data in Table 5 clearly showed that the amount of β-*O*-4′ in the recovered EOL samples decreased. Moreover, the S/G ratios were estimated to be 0.95, 1.06, 0.90, and 0.94 for MWL_u_, MWL_p_, EOL, and CEL, respectively. Similarly as observed by Py-GC/MS of the raw bamboo material and pretreated bamboo, the S/G ratio of MWL_u_ was lower than that of MWL_p_, indicating a decrease of H and G units and an increase of S lignin units during ethanol organosolv treatment [31]. Moreover, the S/G ratio from HSQC NMR spectra was higher than that estimated from Py-GC/MS, corroborating the same observation recently reported by Li *et al.* [32]. However, the results demonstrate that those methods yield relatively similar trends of S/G ratio.

## 3. Experimental Section

### 3.1. Materials

Three year old bamboo (*Dendrocalamus brandisii*) was harvested from Yunnan Province, in the southeast of China. The bamboo was manually chipped and smashed before use. The powder obtained was screened to get particles sized in 40–60 mesh. Subsequently, they were extracted with toluene/ethanol (2:1, *v*/*v*) in a Soxhlet apparatus for 8 h. The cellulolytic enzymes used in this study were Celluclase 1.5 L and Ultraflo L (Novozymes, Tianjin, China) with activities of 700 EGU/g and 45 FBG/g, respectively. Dimethyl sulfoxide-*d*_6_ (DMSO-*d*_6_) was obtained from Aldrich (St. Louis, MO, USA). For analysis, deionized (DI) water was obtained by passing distilled water through a filter apparatus (Pall Corporation, Port Washington, NY, USA). Unless otherwise stated, reagents were purchased from Beijing Chemicals (Beijing, China), and were analytical grade and used as received.

### 3.2. Isolation of Lignins

**Figure 6:**
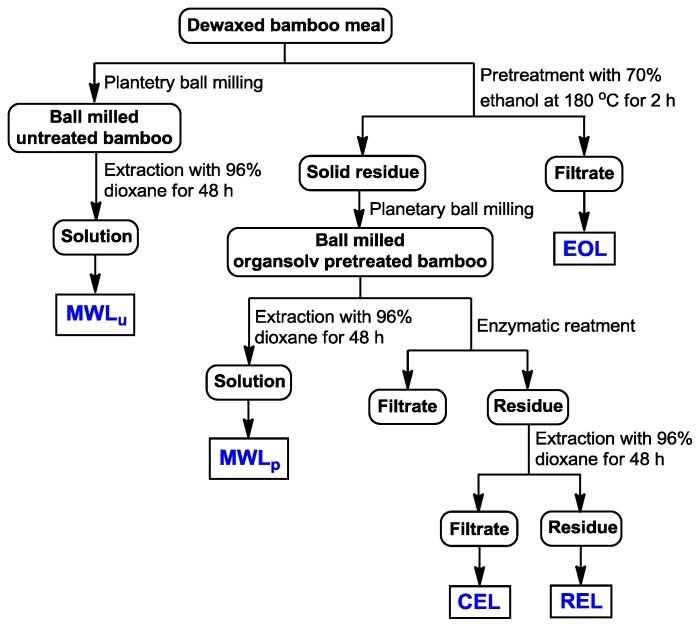
Scheme for the extraction of EOL, MWL, CEL, and REL from bamboo.

The fractionation sequence of the lignin fractions is schematically illustrated in Figure 6. Bamboo sample was pretreated by ethanol organosolv using 70% (*v*/*v*) aqueous ethanol solution at 180 °C for 2 h with a solid to liquid ratio of 1:10 (1 g solid and 10 g liquid) in a 1.0 L pressure reactor with a temperature controller (Parr Instrument Company, Moline, IL, USA). The pretreated bamboo was filtered and dried. After filtration, the filtrate was concentrated to 40 mL under reduced pressure at 50 °C. EOL was obtained by precipitation at pH 2.0 with 6 M HCl and collected by centrifugation as well as freeze-drying.

MWL was isolated from the raw and pretreated bamboo sample according to the method described by Björkman [33]. The samples were firstly milled using a planetary ball milling (Fritsch, Idar-Oberstein, Germany) in a 500 mL ZrO_2_ bowl with mixed balls, 10 balls of 2 cm diameter and 25 balls of 1 cm diameter. The milling was run under a nitrogen atmosphere at 500 rpm with 10 min of rest after every 10 min of milling. Five hours of milling was performed to minimize the structural changes of lignin caused by ball milling. The milled materials were extracted twice with *p*-dioxane-water solution (96% *v*/*v*) in a shaker for 48 h in the dark, respectively. The *p*-dioxane-water extracts were combined and the solvent volume was reduced to about 40 mL using a rotary evaporator (Shanghai Ya Rong Biochemical Instrument Factory, Shanghai, China). Then this solution was added dropwise to deionized (DI) water (200 mL) while stirring and then freeze-dried. The crude MWL was dissolved in 90% acetic acid (20 mL) and precipitated in DI water (400 mL). The solution was centrifuged and the solid part was dissolved in 1,2-dichloroethane/ethanol (10 mL, 2:1 *v*/*v*) and precipitated in diethyl ether (200 mL). Subsequently, the solution was centrifuged and the solid material was washed with petroleum ether (2 × 100 mL). The lignin sample obtained was freeze-dried, referred as MWL_u_ and MWL_p_ respectively. The final yield was around 3%–5% of the original lignin content.

CEL was isolated according to the method described as Chang *et al.* [13] with minor modification. Briefly, 10 g of pretreated sample was incubated twice in acetate buffer (100 mL, pH 4.8) with 20 mL Ultraflo L enzyme and 10 mL of cellulase at 50 °C for 24 h. The reaction system was centrifuged, the supernatant was removed, and the residue was again suspended in acetate buffer (50 mL, pH 4.8) and treated with Ultraflo (10 mL) and cellulase (5 mL) for additional 24 h at 50 °C. After filtration, the enzyme-treated residue was treated by extractions (2 × 24 h) with dioxane/water (100 mL, 96:4, *v*/*v*). The solution was collected by centrifugation and concentration. The crude CEL was freeze-dried and purified as MWL. The residue after CEL isolation was freeze-dried and named as residual enzyme lignin (REL).

### 3.3. Chemical Composition Analysis

The chemical composition of the untreated and pretreated bamboo samples and the lignin samples were determined according to National Renewable Energy Laboratory (NREL) standard analytical laboratory procedure [34]. Briefly, samples (~300 mg) were hydrolyzed with 72% H_2_SO_4_ for 1 h at 30 °C followed by high temperature hydrolysis at 121 °C for 1 h after dilution to 4% H_2_SO_4_. After hydrolysis, the samples were diluted and quantified with High Performance Anion Exchange Chromatography with Pulsed-Amperometric Detection (HPAEC-PAD) on a Dionex ICS3000. Separation was achieved with a CarboPac™ PA-20 analytical column (3 × 150 mm, Dionex, Sunnyvale, CA, USA) and a CarboPac™ PA-20 guard column (3 × 30 mm, Dionex, Sunnyvale, CA, USA). Neutral sugars and uronic acids were separated in isocratic 5 mM NaOH (carbonate-free and purged with nitrogen) for 20 min, followed by a 0.75 mM NaAc gradient in 5 mM NaOH for 15 min with a flow rate of 0.4 mL/min. Calibration was performed with standard solutions of sugars, and the relative standard deviation of the results was below 6%. Ash content was determined by burning the material in an oven at 600 °C according to the method of NREL/TP-510-42622 [35].

### 3.4. Analytical Pyrolysis

Analytical Py-GC/MS of the raw and the pretreated bamboo (about 100 μg) were performed with a CDS Pyroprobe 5200HP pyrolyser autosampler (Chemical Data Systems, Oxford, PA, USA) attached to a PerkinElmer GC/MS apparatus (Clarus 560, PerkinElmer, Waltham, MA, USA) using a 30 × 0.25 mm column (film thickness 0.25 μm). The pyrolysis was carried out into a glass liner at 500 °C for 4 s with the heating rate of 20 °C/ms. The chromatograph was programmed from 40 °C (3 min) to 300 °C at a rate of 6 °C/min. Helium was used as the carrier gas with a constant flow rate of 1 mL/min and a 1:80 split ratio. The mass spectrometer was operated in EI mode (70 eV) and the mass spectra were obtained from *m*/*z* 20 to 400. The injector temperature was kept at 300 °C, while the GC/MS interface was kept at 280 °C [36]. The compounds were identified by comparison with those reported in the literature and in the Wiley and NIST computer libraries [37–39]. Relative peak molar areas (obtained by dividing the peak area by the molecular weight) were calculated for each lignin pyrolysis products. The syringyl/guaiacyl (S/G) ratio was calculated by dividing the sum of peak areas from the sum of the peak areas of syringyl units and by the sum of guaiacyl derivatives of the selected markers, obtained by integration of the peak areas and considering the total peak area as 100%.

### 3.5. FT-IR Analysis

FT-IR spectra were obtained on a spectrophotometer (Nicolet iN10 FT-IR Microscope, Thermo Fisher Scientific, Waltham, MA, USA)) equipped with a liquid nitrogen cooled mercury cadmium telluride (MCT) detector in the range 4000–650 cm^−1^ at 4 cm^−1^ resolution and 128 scans per sample.

### 3.6. Molecular Weight Analysis

Molecular weights of the lignin fractions were measured by GPC with an ultraviolet detector (UV) (Agilent Technologies, Santa Clara, CA, USA) at 240 nm on a PL-gel 10 mm Mixed-B 7.5 mm i.d. calibrated with PL polystyrene. The calibration curve was created by fitting a polynomial equation to the retention volumes obtained from a series of narrow molecular weight distribution polystyrene standards. The samples were acetylated with acetic anhydride before determination according to the literature [27,40] with mild modification. Namely, about 20 mg of dry lignin sample was dissolved in a 1:1 mixture of acetic anhydride/pyridine (1 mL) and stirred at room temperature in darkness for 24 h. Ethanol (25 mL) was added to the reaction mixture, left for 30 min, and then removed with a rotary evaporator. The addition and removal of ethanol was repeated several times until all traces of acetic acid were removed from the lignin sample. The residue was dissolved in chloroform (2 mL) and added to diethyl ether (100 mL). Then the obtained solution was centrifuged. Subsequently, the precipitate was washed three times with diethyl ether and dried in a vacuum over as the acetylated lignin. The derivatized lignin was dissolved in tetrahydrofuran (THF) (1 mg/mL), and the solution was filtered through a 0.45 μm filter. The filtered solution (20 μL) was injected into the HPLC system and the eluted compounds were detected using an UV detector set at 280 nm [41].

### 3.7. NMR Spectra

All NMR spectra were recorded on a Bruker AVIII spectrometer (400M Hz) (Bruker, Zurich, Switzerland) equipped with a *z*-gradient triple resonance probe at 100 MHz in DMSO-*d*_6_. 20 mg of the sample was dissolved in 1 mL DMSO-*d*_6_. The spectral widths were 5000 and 25625 HZ for the ^1^H–^13^C dimensions, respectively, and the numbers of collected complex points were 2048 for the ^1^H dimensions with a recycle delay of 5 s. The number of transients was 64, and 256 time increments were always recorded in the ^13^C-dimensions. The ^1^J_CH_ was set to 146 Hz. Prior to Fourier transform the data matrices were zero filled up to 1024 points in the ^13^C-dimensions. Signals were assigned by comparison to literature spectra. The C–H correlations from S and G type units in the aromatic region were used to estimate the S/G ratio of lignin and the percentage of oxidized units.

## 4. Conclusions

During ethanol organosolv pretreatment, the main degraded compounds are lignin, hemicelluloses, and less ordered cellulose, while leaving most of the ordered cellulose undigested. Furthermore, in this process, the G lignin moiety was preferably degraded as indicated by solid-state NMR and Py-GC/MS. It was found that the milled wood lignin extracted from the original bamboo was HGS lignin with G > S >> H. The spectroscopic results suggested that the ethanol organosolv treatment of the bamboo material predominantly involved the cleavage of β-aryl ether bonds. The lower molecular weight of EOL demonstrated that this process degraded the lignin to a noticeable extent while HSQC NMR and FT-IR spectra showed that the process did not strongly affect lignin primary structures.

## Supplementary Information



## Figures and Tables

**Table 1:** Results of component analysis of the original and pretreated bamboo, and the carbohydrate analysis of the isolated lignin samples (%).

Chemical composition		Untreated bamboo		Pretreated bamboo		
Cellulose (as glucan)		47.24 ± 1.15		52.34 ± 0.32		
Hemicellulose sugars		23.85 ± 1.79		23.41 ± 0.03		
Xylan		22.12 ± 1.53		22.53 ± 0.03		
Arabinan		1.24 ± 0.23		0.68 ± 0.00		
Galactan		0.47 ± 0.05		0.20 ± 0.00		
Mannan		0.07 ± 0.00		ND		
Galacturonic acid		0.03 ± 0.01		ND		
Klason lignin		23.84 ± 1.09		17.27 ± 0.11		
Acid-soluble lignin		1.51 ± 0.06		1.06 ± 0.02		
Ash		1.37 ± 0.03		1.16 ± 0.06		
Samples	Carb a	Ara a	Gal a	Glc a	Xly a	Man a
MWL_u_	10.51 ± 0.25	0.22 ± 0.05	0.10 ± 0.01	6.68 ± 0.05	3.49 ± 0.07	Trace
MWL_p_	8.11 ± 0.87	0.04 ± 0.00	0.02 ± 0.00	6.63 ± 0.71	1.43 ± 0.15	Trace
EOL	5.26 ± 0.42	0.58 ± 0.05	0.32 ± 0.02	1.01 ± 0.11	3.35 ± 0.22	Trace
CEL	12.24 ± 1.24	0.83 ± 0.01	0.49 ± 0.02	5.17 ± 0.12	4.79 ± 0.92	0.95 ± 0.22
REL	24.96 ± 1.38	0.46 ± 0.03	0.92 ± 0.05	17.32 ± 0.91	4.47 ± 0.30	1.47 ± 0.07

a Carb, carbohydrate; Ara, arabinan; Gal, galactan; Glc, glucan; Man, mannan; Xyl, xylan.

**Table 2:** Composition, retention time, formula, molecular weight (M_w_) and relative molar abundance (%) of the compounds released after Py-GC/MS of bamboo.

Label	R.T. (min)	Compound name	Formula	M*_w_*	Untreated	Pretreated
**Carbohydrate derived compounds**						
1	2.82	2-Cyclopenten-1-one, 2-hydroxy-	C_5_H_6_O_2_	98	0.9	1.6
2	4.63	2-Hydroxy-gamma-butyrolactone	C_4_H_6_O_3_	102	6.5	2.2
3	5.88	Furan, 2,5-dimethyl-	C_6_H_8_O	96	3.3	2.2
4	6.49	2-Furanmethanol	C_5_H_6_O_2_	98	0.8	0.5
5	7.97	2(5*H*)-furanone	C_4_H_4_O_2_	84	2.3	0.6
6	8.42	2-Cyclopenten-1-one, 2-hydroxy-	C_5_H_6_O_2_	98	4.5	2.3
8	10.89	2-Cyclopenten-1-one, 2-hydroxy-3-methyl-	C_6_H_8_O_2_	112	0.3	0.6
9	11.20	1,2-Cyclopentanedione, 3-methyl-	C_6_H_8_O_2_	112	1.8	0.6
13	13.87	2,4(3H,5H)-furandione, 3-methyl-	C_5_H_6_O_3_	114	1.4	1.1
17	16.38	2-Furancarboxaldehyde, 5-(hydroxymethyl)-	C_6_H_6_O_3_	126	1.1	2.3
28	22.67	β-D-Glucopyranose, 1,6-anhydro-	C_6_H_10_O_5_	162	21.0	65.8
	Sum				43.9	79.9
**Lignin guaiacyl-type**						
10	12.00	Phenol, 2-methyl-	C_7_H_8_O	108	1.2	0.4
12	12.85	Phenol, 2-methoxy-	C_7_H_8_O_2_	124	1.5	0.4
14	15.49	Phenol, 2-methoxy-4-methyl-	C_8_H_10_O_2_	138	0.9	0.3
15	15.59	1,2-Benzenediol	C_6_H_6_O_2_	110	1.8	0.5
20	17.80	1,2-Benzenediol, 4-methyl-	C_7_H_8_O_2_	124	0.9	0.5
21	18.37	2-Methoxy-4-vinylphenol	C_9_H_10_O_2_	150	2.8	0.9
24	20.25	Vanillin	C_8_H_8_O_3_	152	1.1	0.4
25	21.23	1,2,4-Trimethoxybenzene	C_9_H_12_O_3_	168	0.7	0.4
26	21.37	Phenol, 2-methoxy-4-(1-propenyl)-	C_10_H_12_O_2_	164	1.2	0.4
27	22.07	Ethanone, 1-(4-hydroxy-3-methoxyphenyl)-	C_9_H_10_O_3_	166	1.6	0.3
30	22.92	2-Propanone, 1-(4-hydroxy-3-methoxyphenyl)-	C_10_H_12_O_3_	180	0.4	Trace
36	25.27	2-Butanone, 4-(4-hydroxy-3-methoxyphenyl)-	C_11_H_14_O_3_	194	0.5	0.2
40	26.84	2-Propenal, 3-(4-hydroxy-3-methoxyphenyl)-	C_10_H_10_O_3_	178	1.6	0.2
41	26.94	4-((1E)-3-Hydroxy-1-propenyl)-2-methoxyphenol	C_10_H_12_O_3_	180	6.4	0.4
	Sum				22.7	5.3
**Lignin syringyl-type**						
18	17.08	1,2-Benzenediol, 3-methoxy-	C_7_H_8_O_3_	140	1.1	0.3
22	19.17	Phenol, 2,6-dimethoxy-	C_8_H_10_O_3_	154	1.9	0.4
29	22.82	Benzene, 1,2,3-trimethoxy-5-methyl-	C_10_H_14_O_3_	182	0.5	Trace
32	23.63	3′,5′-Dimethoxyacetophenone	C_10_H_12_O_3_	180	2.4	0.7
37	25.43	Benzaldehyde, 4-hydroxy-3,5-dimethoxy-	C_9_H_10_O_4_	182	0.9	0.4
38	26.25	Phenol, 2,6-dimethoxy-4-(2-propenyl)-	C_11_H_14_O_3_	194	1.6	0.5
39	26.76	Ethanone, 1-(4-hydroxy-3,5-dimethoxyphenyl)-	C_10_H_12_O_4_	196	0.7	0.3
43	30.25	3,5-Dimethoxy-4-hydroxycinnamaldehyde	C_11_H_12_O_4_	208	0.6	0.4
	Sum				9.7	3.1
**Other lignin derived products**						
7	10.01	Phenol	C_6_H_6_O	94	1.4	0.5
11	12.57	Phenol, 3-methyl-	C_7_H_8_O_2_	108	1.0	0.5
16	16.19	4-Methyl-benzaldehyde	C_8_H_8_O	120	10.0	2.6
19	17.52	Hydroquinone	C_6_H_6_O_2_	110	0.9	0.2
23	19.47	Benzaldehyde, 4-hydroxy-	C_7_H_6_O_2_	122	0.7	0.3
31	23.31	4-Acetylbenzoic acid	C_9_H_8_O_3_	164	0.9	1.2
33	23.69	1,4-Benzenedicarboxylic acid, methyl ester	C_9_H_8_O_4_	180	0.9	1.3
34	24.07	trans-Cinnamic acid	C_9_H_8_O_2_	148	0.3	0.1
35	25.07	1,4-Benzenedicarboxylic acid	C_8_H_6_O_4_	166	0.9	0.6
	Sum				16.8	7.4
	S/G				0.4	0.6

**Table 3:** Weight average (M_w_) and number average (M_n_) molecular weights and dispersity (M_w_/M_n_) index of the acetylated fractionated lignin samples.

Heading	MWL_u_	MWL_p_	EOL	CEL
M*_w_* (g/mol)	7692	10657	5873	15307
M*_n_* (g/mol)	4406	5997	3072	9721
M*_w_*/M*_n_*	1.75	1.78	1.91	1.57

**Table 4:** Assignments of main lignin and polysaccharide ^13^C–^1^H correlation signals in the HSQC spectra of lignin fractions from bamboo *D. brandisii* shown in Figure 4.

Labels	δ_C_/δ_H_ (ppm)	Assignment
Lignin cross-signals		
C_β_	53.2/3.43	C_β_–H_β_ in β-5′ (phenylcoumaran) substructures (C)
B_β_	53.5/3.05	C_β_–H_β_ in β-β′ (resinol) substructures (B)
–OMe	55.9/3.72	C–H in methoxyls (MeO)
A_γ_	60.1/3.22 and 59.67/3.59	C_γ_–H_γ_ in β-*O*-4′ substructures (A) and others
I_γ_	61.4/4.09	C_γ_–H_γ_ in cinnamyl alcohol end-groups (I)
A′_γ_	62.8/4.28	C_γ_–H_γ_ in γ-acylated β-*O*-4′ substructures (A′)
C_γ_	62.4/3.71	C_γ_–H_γ_ in β-5′ (phenylcoumaran) substructures (C)
I′_γ_	64.1/4.77	C_γ_–H_γ_ in γ-acylated cinnamyl alcohol end-groups (I′)
A_α_	72.4/4.85	C_α_–H_α_ in β-*O*-4′ substructures (A)
B_γ_	71.0/4.17 and 70.9/3.80	C_γ_–H_γ_ in β-β′ (resinol) substructures (B)
A_β_(G)	83.6/4.30	C_β_–H_β_ in β-*O*-4′ substructures linked to a guaiacyl unit (A)
C_α_	87.1/5.45	C_α_–H_α_ in β-5′ (phenylcoumaran) substructures (C)
A_β_(S)	85.8/4.10	C_β_–H_β_ in β-*O*-4′ substructures linked to a syringyl unit (A, *erythro*)
A_β_(S)	86.2/3.99	C_β_–H_β_ in β-*O*-4′ substructures linked to a syringyl unit (A, *threo*)
B_α_	84.7/4.65	C_α_–H_α_ in β-β′ (resinol) substructures (B)
T′_2/6_	103.9/7.32	C′_2,6_–H′_2,6_ in tricin (T)
T_3_	106.1/7.04	C_3_–H_3_ in tricin (T)
T_6_	98.8/6.22	C_2,6_–H_2,6_ in tricin (T)
S_2/6_	104.3/6.70	C_2,6_–H_2,6_ in syringyl units (S)
S′_2/6_	106.3/7.30	C_2,6_–H_2,6_ in oxidized (C_α_OOH) syringyl units (S′)
G_2_	111.1/6.97	C_2_–H_2_ in guaiacyl units (G)
G_5_	115.8/6.69	C_5_–H_5_ and C_6_–H_6_ in guaiacyl units (G)
**G_6_**	119.1/6.79	C_6_–H_6_ in guaiacyl units (**G**)
**PCA_7_**	144.5/7.43	C_7_–H_7_ in *p*-coumaroylated substructures (**PCA**)
**PCA_2/6_**	130.2/7.46	C_2.6_–H_2.6_ in *p*-coumaroylated substructures (**PCA**)
**PCA_3/5_**	115.4/6.76	C_3_–H_3_ and C_5_–H_5_ in *p*-coumaroylated substructures (**PCA)**
**PCA_8_**	113.6/6.26	C_8_–H_8_ in *p*-coumaroylated substructures (**PCA**)
**FA_2_**	111.5/7.49	C_2_–H_2_ in ferulate (**FA**)
**H_2/6_**	128.0/7.17	C_2.6_–C_2.6_ in *p*-hydroxyphenyl units (**H**)
**H_3/5_**	115.2/6.57	C_3.5_–C_3.5_ in *p*-hydroxyphenyl units (**H**)
**J_α_**	153.5/7.61	C_α_–H_α_ in cinnamyl aldehyde end-groups (**J**)
**J_β_**	126.2/6.79	C_β_–H_β_ in cinnamyl aldehydes end-groups (**J**)
**D′_β_**	80.3/4.54	C′_β_–H′_β_ in spirodienone substructure (**D**)
Polysaccharide cross-signals		
**X_2_**	70.1/3.33	C_2_–H_2_ in β-d-xylopyranoside
**X_3_**	72.0/3.42	C_3_–H_3_ in β-d-xylopyranoside
**X_4_**	75.3/3.54	C_4_–H_4_ in β-d-xylopyranoside
**X_5_**	62.8/3.40	C_5_–H_5_ in β-d-xylopyranoside

**Table 5:** Structural characteristics (lignin interunit linkages, relative molar composition of the lignin aromatic units, S/G ratio and *p*-coumarate/and ferulate content and ratio) from integration of C–H correlation signals in the HSQC spectra of the isolated lignin fractions.

	MWL_u_ (%)	MWL_p_ (%)	EOL (%)	CEL (%)
Lignin interunit linkages				
β-*O*-4′ substructure (A)	89.4	82.1	72.3	94.5
β-β′ resinol substructures (B)	5.5	2.6	20.0	0
β-5′ phenylcoumaran substructures (C)	5.1	15.3	7.7	5.5
Lignin aromatic units				
H	3.5	–	19.6	8.0
G	49.5	48.5	42.4	47.5
S	47.0	51.5	38.0	44.5
S/G ratio	0.95	1.06	0.90	0.94
*p*-Hydroxycinnamates				
*p*-Coumarates	97.5	84.9	82.1	76.6
Ferulates	9.3	15.1	17.9	23.4
*p*-Coumarates/ferulates ratio	9.75	5.62	4.59	3.27
