# Supplementary material for: Structural Variation of Bamboo Lignin before and after Ethanol Organosolv Pretreatment
Source: Int J Mol Sci. 2013 Oct 28;14(11):21394–413. doi: 10.3390/ijms141121394 (PMC3856011; doi:10.3390/ijms141121394)
Supplement: Supplementary file 1 [file ijms-14-21394-s001.pdf]

Supplementary Information

Table S1. Assignments of the FT-IR spectra of the lignin samples.

Assignments	Wavenumbers (cm^{-1})
O–H stretch	3401
C–H stretch in methyl and methylene groups	2939
C=O stretch in unconjugated ketones, carbonyl and in ester groups (frequently of carbonhydrate origin)	1705
C=O stretch; in conjugated *p*-substituted aryl ketones	1655
aromatic skeletal vibrations; S > G	1593
C-H deformations; asym. in $–CH_3$ and $–CH_2–$	1504
Aromatic skeletal vibrations combined with C-H in-plane deform	1423
Aliphatic C-H stretch in CH_3, not in OMe; phen.OH	1362
condensed S and G rings (substituted in one of the positions in the ring)	1327
G ring plus C=O stretch	1261
Typical for HGS lignins; C=O in ester groups (conjugated)	1161
Aromatic C-H in-plane deformation	1122
Aromatic C-H in-plane deformation, G > S; plus C-O deform, in primary alcohols; plus C=O stretch (unconjugated)	1030
C-H out-of-plane in positions 2, 5, and 6 of G units	833

© 2013 by the authors; licensee MDPI, Basel, Switzerland. This article is an open access article distributed under the terms and conditions of the Creative Commons Attribution license (http://creativecommons.org/licenses/by/3.0/).
